# Changes of serum 25(OH) D3 and IGF-1 levels in patients with thyroid nodules

**DOI:** 10.1186/s12902-019-0376-1

**Published:** 2019-05-10

**Authors:** Xueqin Du, Yi Liu, Chunhui Zhao, Jingzhou Fang, Xiangna Wang, Limin Wei

**Affiliations:** 1 Hebei Medical University, Shijiazhuang, 050017 Hebei China; 2 Hebei North University, Zhangjiakou, 075000 Hebei China; 3 Department of Pediatrics, Shijiazhuang Fourth Hospital, Shijiazhuang, 050011 Hebei China; 4 Department of Endocrinology, Hebei General Hospital, Shijiazhuang, 050051 Hebei China

**Keywords:** Thyroid nodules, 25(oh)D3, Insulin-like growth factor-1, Deficiency of vitamin D, Correlation analysis

## Abstract

**Background:**

The present study aimed to study the relationship between serum 25 hydroxyvitamin D3(25(OH)D3) and insulin-like growth factor-1 (IGF-1) and thyroid nodules.

**Methods:**

Two hundred eighty-nine cases with thyroid nodules and 109 health subjects (control group) who admitted to the Hebei General Hospital during June 2016 to December 2016 were included in the study. Basic clinical information (age, sex, thyroid function, liver and kidney function, hypertension history, etc.) of patients were collected. Serum 25(OH) D3 and Serum IGF-1 were detected by electrochemiluminescence and radioimmunoassay methods, respectively. The relationship between the above-mentioned factors and thyroid nodules was statistically analyzed.

**Results:**

Serum 25(OH)D3, IGF-1, fasting blood glucose (FBG), total cholesterol (TC), waist circumference (WC), total triiodothyronine (TT3), total thyroxine (TT4), hypertension history, and drinking history were significantly different between the nodules group and the control group (*P < 0.05*). Logistic regression analysis showed that there was a negative correlation between thyroid nodules and levels of 25(OH)D3, IGF-1, TT3, as well as a positive correlation with FBG, TC, TT4, and hypertension. There was a positive correlation between IGF-1 and serum 25(OH)D3 in thyroid nodules (*P* < 0.05). After correcting the aforementioned factors, high-level of serum 25(OH)D3 was significantly correlated with the decreased incidence of thyroid nodules.

**Conclusions:**

The incidence of thyroid nodules is relatively lower in a high-level of serum 25(OH)D3, and serum 25(OH)D3 may be a direct protective factor for thyroid nodules. Serum IGF-1 can be one of the indirect protective factors for thyroid nodules as well.

**Electronic supplementary material:**

The online version of this article (10.1186/s12902-019-0376-1) contains supplementary material, which is available to authorized users.

## Background

Thyroid nodules is an independent lesion in the thyroid gland, and it is one of the common diseases on the endocrine system. With the broad application of high frequency ultrasound scanning, the detection rate of thyroid nodules in healthy population has reached as high as 50–60% [1], and that is more common in female and elderly people as well. With the popularization of health examination and the appearance of thyroid color Doppler as a general examination item, the detection rate of several asymptomatic thyroid nodules is increasing, and individuals pay further attention to it. At present, the pathogenesis of thyroid nodules is not fully clear.

The lack of vitamin D3 is closely associated with the development of a variety of diseases such as, cardiovascular disease and malignant tumor. However, the relationship between the changes of serum vitamin D3 levels and the thyroid nodules is not still clear. Roskies et al. [2] found that the lack of serum 25(OH)D3 may be associated with the occurrence of thyroid cancer. IGF-1 probably plays an important role in the genesis and development of certain solid cold thyroid nodules, including papillary thyroid carcinomas, nodular goiters, and follicular adenomas [3]. High 25(OH)D3 level may be a factor in reducing cell proliferation and inducing apoptosis through complementary pathways or mechanisms.

Based on the above-mentioned researches, the purpose of this study is to further clarify the changes and interactions of serum 25(OH)D3 and IGF-1 levels in patients with thyroid nodules. The results provide further theoretical basis for the study of the mechanism of thyroid nodule development.

## Methods

### Subjects

Here, 410 health examination subjects were collected in Hebei General Hospital (China) during June 2016 to December 2016 with ultrasound examination. Among them, 300 cases found with thyroid nodules, while 110 cases did not have thyroid nodules. Exclusion criteria: thyroid nodules demonstrated malignant tendency of thyroid ultrasound, abnormality of thyroid function, thyroid related diseases, liver and kidney diseases, diabetes, malignancies, acromegaly, and subjects with the history of acute disease. The final subjects included 289 cases with thyroid nodules (nodule group) and 109 cases as normal subjects (control group). All subjects did not take therapies (LT4, cholecalciferol, etc.) that could affect the parameters considered.

### Methods

#### Collection of general data

All participants were asked to fill in questionnaires designed by professionals (The questionnaire see in Additional file 1). General information covered sex, age, hypertension history, smoking, and drinking. All the subjects were tested based on the body parameters by the same group of operators, including height, weight, waist circumference, blood pressure, and body mass index (BMI).

#### Ultrasound examination

Ultrasound examination of thyroid nodules was using PHILIPS EPIQ7 color Doppler ultrasound system, L12–5 probe, frequency 7~12 MHz. The size of thyroid nodules was recorded, the ultrasonic data of thyroid nodules were quantified, and the cross-sectional area of thyroid nodules was calculated as well.

#### Detection of metabolic parameters

Biochemical tests were performed on fasting blood for 8–10 h. The main factors including fasting blood glucose (FBG), total cholesterol (TC), triglyceride (TG), high density lipoprotein cholesterol (HDL-C), low density lipoprotein cholesterol (LDL-C) were analyzed by an automatic biochemical analyzer, and serum 25(OH)D3, fasting insulin (FINS) and thyroid function were measured by electrochemiluminescence, and serum IGF-1 was determined by radioimmunoassay.

### Statistical analysis

Data obtained from the study were analyzed using SPSS 19.0 software (SPSS Inc., IL, USA). The normal distribution of measurement data was expressed by mean ± standard deviation ($\overline{x}$ ±s), and non-normal state of measurement data is expressed by median (four quantiles). Two samples were normal and the variance was homogeneous compared with *t-*test, otherwise, a non-parametric test needed to be applied. Comparing the differences between two or more overall rates or the correlation between categorical variables were performed by *χ*^*2*^ test. A linear correlation analysis between two variables was carried out using *Spearman*’s correlation test. Two correlation analyses between categorical variables and some effective factors were undertaken using Logistic Regression Analysis. (LRA) *P-value* < 0.05 was statistically considered significant.

## Results

### Comparing the general data between the nodular group and the control group

The gender, age, 25(OH)D3, IGF-1, FBG, blood lipid, thyroid function, liver function, and other factors in the nodular group and the control group were compared. The results showed that the serum 25(OH)D3 level in the nodular group was lower than that in the control group (9.86 ± 6.66 vs. 12.74 ± 7.13 ng/L) (*P < 0.001*); the serum IGF-1 level in the nodular group was lower than that in the control group (111.10(81.53, 165.44) vs. 192.65(96.38, 313.49) ng/L), and the differences were statistically significant (*P < 0.001*) (Table 1). The FBG, TC, WC, TT3, TT4, and hypertension and drinking histories were different between the two groups (*P < 0.05*).

**Table 1:** Difference analysis of basic clinical information between the nodule group and control group

	Thyroid nodules	Control	*P* value
n	289	109	
Age(year)	46(40,53)	45(40,49.5)	0.351
Sex			0.498
Male	158(54.67)	64(58.72)	
Female	131(45.33)	45(41.28)	
ALT(U/L)	25.31 ± 19.57	26.38 ± 17.20	0.621
AST(U/L)	21.03 ± 9.03	20.83 ± 7.10	0.841
r-GT(U/L)	34.73 ± 36.59	32.46 ± 29.49	0.562
Uric(umol/L)	314.39 ± 84.70	318.06 ± 78.24	0.694
FBG(mmol/L)	5.42(5.07,5.94)	5.20(4.92,5.43)	< 0.001
TC(mmol/L)	5.17 ± 0.93	4.62 ± 0.86	< 0.001
TG(mmol/L)	1.25(0.84,1.99)	1.13(0.81,1.69)	0.195
HDL-C(mmol/L)	1.29 ± 0.33	1.30 ± 0.29	0.764
LDL-C(mmol/L)	2.58 ± 0.65	2.54 ± 0.66	0.628
BMI(kg/m^2^)	25.63 ± 3.37	25.04 ± 3.70	0.129
WC(cm)	88.19 ± 10.44	85.60 ± 11.41	0.032
Smoking			0.239
Never	197 (68.17)	83(76.15)	
Former	16(5.54)	3(2.75)	
Current	76(26.29)	23(21.10)	
Drinking			0.021
Never	151(52.25)	48(44.034)	
Former	7(2.42)	1(0.92)	
Sometimes	63(21.80)	40(36.70)	
Often	68(23.53)	20(18.34)	
TT3(pmol/L)	1.96 ± 0.35	2.09 ± 0.27	< 0.001
TT4(pmol/L)	111.62 ± 20.16	98.42 ± 15.19	< 0.001
TSH(uIU/ml)	1.89 ± 1.72	2.00 ± 0.98	0.366
TPOAb(IU/ml)	10.54(6.82,15.89)	11.74(8.23,16.85)	0.139
FINS(uU/ml)	9.93 ± 7.61	9.95 ± 5.26	0.971
25(OH)D3(ng/L)	9.86 ± 6.66	12.74 ± 7.13	< 0.001
IGF-1(ng/ml)	111.10(81.53,165.44)	192.65(96.38,313.49)	< 0.001
HOMA-IR	1.96(1.25,3.19)	2.20(1.52,2.82)	0.929
Hypertention	55(19.03)	5(4.59)	< 0.001

### The correlation between the single factors and the thyroid nodules

Thyroid nodule was analyzed by LRA to reveal the effects of the single factors on the thyroid nodules, including sex, age, 25(OH)D3, IGF-1, FBG, blood lipid, WC, uric acid, thyroid function, liver function, BMI, HbA1c, HOMA-IR, hypertension, smoking history, and drinking history. The variables included FBG, TC, TT3, TT4, hypertension, 25(OH)D3, and IGF-1. The *χ*^*2*^ value of LRA is 148.037 (*P < 0.05*), so the LRA has statistical significance. Therefore, 25(OH)D3, IGF-1 and TT3 were negatively correlated with thyroid nodules, while FBG, TC, TT4, and hypertension were positively correlated with thyroid nodules (Table 2).

**Table 2:** The correlation results between the single factors and thyroid nodules

Dependent variables	Independent variables	B	OR	95% CI	*P* value
Thyroid nodules	FBG	0.610	1.840	1.123–3.015	0.016
	TC	0.418	1.518	1.086–2.127	0.015
	TT3	−3.977	0.019	0.005–0.066	< 0.001
	TT4	0.079	1.082	1.058–1.106	< 0.001
	Hypertention	0.661	3.752	1.220–11.534	0.021
	25(OH)D3	−0.047	0.954	0.917–0.991	0.016
	IGF-1	−0.002	0.998	0.996–1.000	0.013

### The correlation of serum 25(OH)D3 and IGF-1 with each factor

The correlation of serum 25(OH)D3 with age, IGF-1, FBG, blood lipid, FINS, thyroid function, liver function, and cross sectional area of thyroid nodules was analyzed by Spearman’s correlation analysis. The results showed that serum 25(OH)D3 was positively correlated with IGF-1(Table 3) (*r* = 0.123, *P* = 0.037), while there was no significant correlation with aforementioned factors.

**Table 3:** The correlation analysis results of serum 25(OH)D3 and various factors

	r	*P* value		r	*P* value
Age(year)	0.030	0.087	BMI(kg/m^2^)	0.022	0.390
ALT(U/L)	− 0.042	0.477	WC(cm)	0.024	0.360
AST(U/L)	−0.069	0.240	TT3(pmol/L)	−0.036	0.539
r-GT(U/L)	0.034	0.570	TT4(pmol/L)	−0.036	0.545
Uric(umol/L)	−0.002	0.974	TSH(uIU/ml)	−0.072	0.219
FBG(mmol/L)	−0.360	0.540	TPOAb(IU/ml)	−0.002	0.968
TC(mmol/L)	0.015	0.804	FINS(uU/ml)	−0.024	0.683
TG(mmol/L)	0.033	0.572	IGF-1(ng/ml)	0.123	0.037
HDL-C(mmol/L)	0.043	0.466	HOMA-IR	−0.020	0.732
LDL-C(mmol/L)	0.071	0.228	Thyroid nodules cross-sectional area(mm^2^)	0.080	0.176

The results of correlation between serum IGF-1 and aforementioned factors showed that the serum IGF-1 was negatively correlated with FBG (Table 4) (*r* = − 0.207, *P < 0.001*), and was positively correlated with 25(OH)D3 (*r* = 0.123, *P* = 0.037), while no significant correlation was found with other factors.

**Table 4:** The correlation analysis results of serum IGF-1 and various factors

	r	*P* value		r	*P* value
Age(year)	0.042	0.481	BMI(kg/m^2^)	− 0.116	0.051
ALT(U/L)	− 0.162	0.057	WC(cm)	−0.096	0.102
AST(U/L)	−0.138	0.089	TT3(pmol/L)	−0.068	0.251
r-GT(U/L)	−0.148	0.109	TT4(pmol/L)	−0.110	0.062
Uric(umol/L)	−0.113	0.055	TSH(uIU/ml)	0.096	0.104
FBG(mmol/L)	−0.207	0.000	TPOAb(IU/ml)	−0.056	0.345
TC(mmol/L)	−0.056	0.345	FINS(uU/ml)	−0.084	0.155
TG(mmol/L)	−0.091	0.122	25(OH)D3(ng/L)	0.123	0.037
HDL-C(mmol/L)	−0.140	0.057	HOMA-IR	−0.023	0.079
LDL-C(mmol/L)	0.000	0.998	Thyroid nodules cross-sectional area(mm^2^)	0.086	0.145

### Correlation between 25(OH)D3 level and incidence of thyroid nodules

**Fig. 1:**
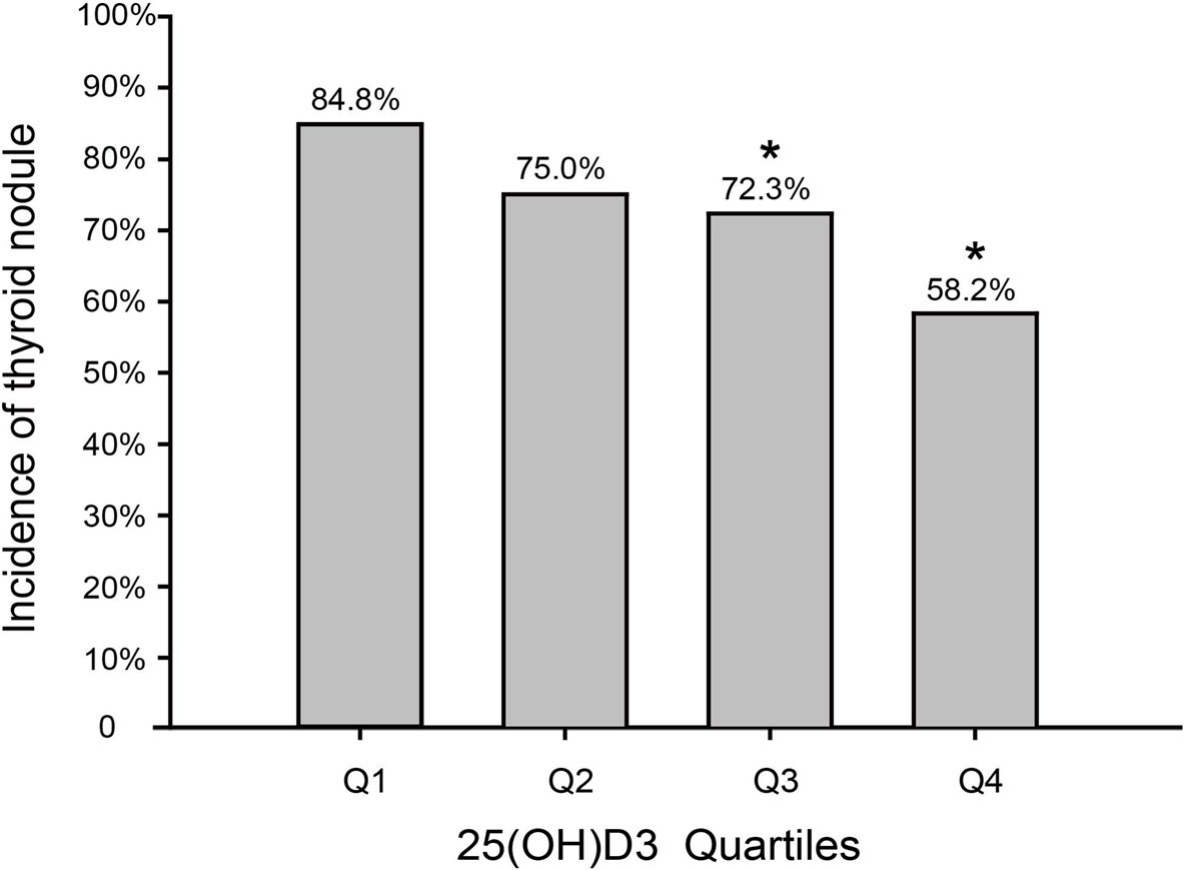
The incidence of thyroid nodules in association with quartiles of serum 25(OH)D3 level. Note: Compared with subjects in the first quartile, a decrease of incidence of thyroid nodules was observed in the second, third, and the fourth quartiles (*P* = 0.083, *P* = 0.030, and *P < 0.001*). *P < 0.05, compared with subjects in the first quartile

According to the quantile of serum 25(OH)D3, all the subjects were divided into four groups: Q1: < 5.19 ng/L (*n* = 99), Q2: 5.20–9.59 ng/L (*n* = 100), Q3: 9.6–14.01 ng/L (*n* = 101), and Q4: ≥ 14.02 ng/L (*n* = 98), and the incidence of thyroid nodules for each group was 84.8, 75, 72.3, and 58.2%, respectively. The results showed that the incidence of thyroid nodules in group Q3 and group Q4 was lower than that in group Q1, and the difference was statistically significant (*P* = 0.030). The incidence of thyroid nodules in group Q2 was lower than that in group Q1, however, the difference was not statistically significant (*P* = 0.083)(Fig. 1).

As LRA showed that IGF-1, TT3, FBG, TC, TT4, and hypertension were also associated with thyroid nodules, correction of the above-mentioned factors (i.e., the correction of different confounding factors through Model 1 (unadjusted), Model 2 (adjusted for FBG, TC, TT3, TT4, and hypertension factors), and Model 3 (further adjusted for IGF-1)) was used to further assess the correlation between the 25(OH)D3 level and the incidence of thyroid nodules (Table 5).

**Table 5:** The correlation between different 25(OH)D3 concentrations and thyroid nodules incidence

	25(OH)D3 quartiles			
	Q1: ≤5.19	Q2: 5.20–9.69	Q3: 9.70–14.01	Q4: ≥14.02
No.cases/subjects	84/99	75/100	73/101	57/98
Model 1	1.00(reference)	0.536(0.263,1.092)	0.628(0.501,0.788)	0.682(0.481,0.969)
Model 2	1.00(reference)	0.493(0.208,1.169)	0.560(0.365,0.860)	0.644(0.478,0.866)
Model 3	1.00(reference)	0.510(0.208,1.249)	0.562(0.365,0.864)	0.622(0.489,0.896)

In Model 1, as a reference for Q1 group, compared with Q3 group, the risk of thyroid nodules decreased (OR = 0.682, 95% CI: 0.481–0.969); after Model 2 corrected FBG, TC, TT3, TT4, and hypertension, the risk of thyroid nodules in Q3 group was still lower than that in Q1 group (OR = 0.560, 95% CI: 0.365–0.860); after further correction of serum IGF-1 by Model 3, the risk of thyroid nodules in Q3 group was still lower than that in Q1 group (OR = 0.562, 95% CI: 0.365–0.864). In Model 1, as a reference for Q1 group, compared with Q4 group, the risk of thyroid nodules decreased (OR = 0.628, 95% CI: 0.501–0.788); after Model 2 corrected FBG, TC, TT3, TT4, and hypertension, the risk of thyroid nodules in Q4 group was still lower than that in Q1 group (OR = 0.644, 95% CI: 0.478–0.866); after further correction of serum IGF-1 by Model 3, the risk of thyroid nodules in Q4 group was still lower than that in Q1 group (OR = 0.662, 95% CI: 0.489–0.896).

### Correlation between IGF-1 level and the incidence of thyroid nodules

**Fig. 2:**
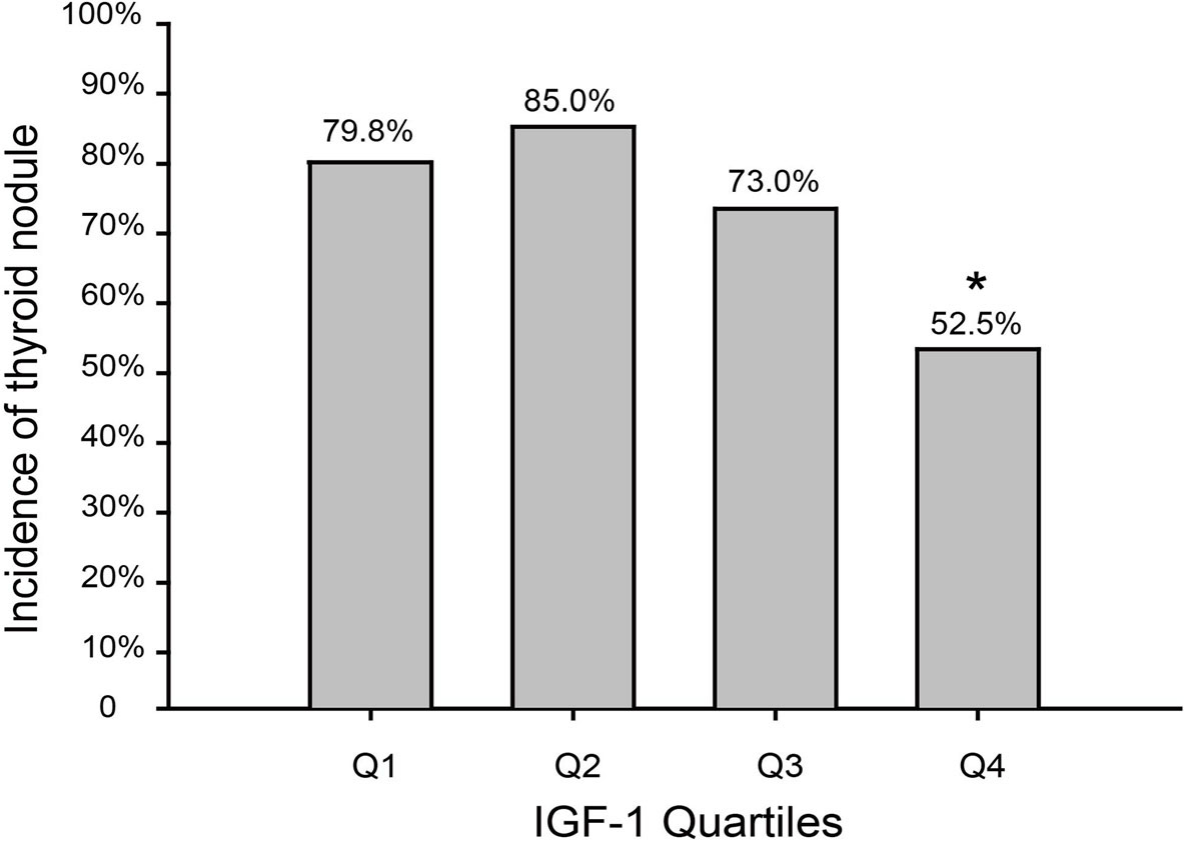
The incidence of thyroid nodules in association with quartiles of serum IGF-1 level. Note: Compared with subjects in the first quartile, a decrease of incidence of thyroid nodules was observed in the second, third, and fourth quartiles (*P* = 0.335, *P* = 0.259, and *P* < 0.001). *P < 0.05, compared with subjects in the first quartile

According to the levels of IGF-1, all the subjects were divided into four groups, namely Q1: < 84.34 ng/L (*n* = 99), Q2: 84.35–120.01 ng/L (*n* = 100), Q3: 120.02–222.30 ng/L (*n* = 100), and Q4: ≥ 222.31 ng/L (*n* = 99). In Q1 group to Q4 group, the incidence of thyroid nodules was 79.8, 85, 73, and 52.5%, respectively. The results showed that compared with Q1 group, the incidence of thyroid nodules decreased in Q4 group, and a significant difference was observed as well (Table 6) (*P < 0.001*), while there was no significant difference between Q2 group and Q3 group compared with Q1 group (Fig. 2).

**Table 6:** The correlation between different IGF-1 concentrations and thyroid nodules incidence

	IGF-1 quartiles			
	Q1: ≤84.34	Q2: 84.35–120.01	Q3: 120.02–222.30	Q4: ≥222.31
No.cases/subjects	79/99	85/100	73/100	52/99
Model 1	1.00(reference)	1.435(0.687,2.995)	0.827(0.595,1.151)	0.654(0.530,0.807)
Model 2	1.00(reference)	1.193(0.468,3.042)	0.709 (0.461,1.092)	0.783(0.607,1.010)
Model 3	1.00(reference)	1.274(0.491,3.3310)	0.756(0.484,1.181)	0.818(0.630,1.063)

Note: Model1 is unadjusted. Model 2 is adjusted for FBG, TC, TT3, TT4, hypertension. Model 3 is further adjusted for 25(OH)D3

LRA showed that 25(OH)D3, TT3, FBG, TC, TT4, and hypertension were associated with thyroid nodules. The above-mentioned factors were also corrected, and the correlation between the level of IGF-1 and the incidence of thyroid nodules was further assessed. Compared with Model 1 as a reference for Q1 group, the risk of thyroid nodules in Q4 group decreased (OR = 0.654, 95% CI: 0.530–0.807). In Model 1, as a reference for Q1 group, compared with Q4 group, the risk of thyroid nodules decreased (OR = 0.654, 95% CI: 0.530–0.807); after Model 2 corrected FBG, TC, TT3, TT4, and hypertension, the risk of thyroid nodules in the Q4 group was still lower than that in the Q1 group, however, the difference was not statistically significant.

## Discussion

Vitamin D is a steroid hormone, and it not only can regulate the metabolism of calcium and phosphorus in the body, but also plays an important role in the occurrence and development of numerous human diseases [4–6], therefore, a great number of individuals tend to consume vitamin D to prevent different diseases [7, 8]. The content of serum 25(OH)D3 in the body is higher than that of serum 1,25(OH) 2D3. Therefore, the serum level of 25(OH)D3 is typically selected to reflect the state of vitamin D in the body. IGF-1 is a peptide hormone composed of 70 amino acids and its amino acid chain is similar to that of the insulin chain [9]. After birth, the IGF-1 produced by the liver is the main source of circulating IGF-1, and the increase of circulating IGF-1 level is more tangible in puberty and adulthood [10]. Numerous studies have shown that the occurrence and development of cardiovascular diseases, malignant tumors, osteoporosis, and other diseases are associated with circulating IGF-1 levels [11, 12]. This study aimes to clarify the changes and interactions of serum 25(OH)D3 and IGF-1 levels in thyroid nodules patients.

Vondra et al.’s study demonstrated that the increase in the risk of autoimmune thyroid disease was associated with deficiency of vitamin D. Serum 25(OH)D3 participates in the pathophysiological process of autoimmune thyroid diseases, and the risk of diffuse toxic goiter (Graves’ disease) is associated with the gene encoding vitamin D binding protein and the polymorphism of 1,25(OH) 2D3 specific receptor remove “target cells” [13]. The relationship between vitamin D and thyroid disease is not still fully clear [14, 15]. This study revealed that the serum 25(OH)D3 level in the nodular group was significantly lower than that in the control group. LRA showed that 25(OH)D3, IGF-1, FBG, TC, TT3, TT4, and high blood pressure were associated with thyroid nodules. Correlation analysis suggested that serum 25(OH)D3 was positively correlated with IGF-1. Therefore, it is presumed that these factors may affect the relationship between serum 25(OH)D3 and the incidence of thyroid nodules. All the subjects were divided into four groups according to the four quantiles of serum 25(OH)D3. The incidence of thyroid nodules was significantly decreased in Q1 group to Q4 group. Compared with the Q1 group, the incidence of thyroid nodules in Q3 group and Q4 group reduced, and there were statistically significant differences (*P < 0.05*). After correcting the aforementioned factors, the risk of thyroid nodules still decreased with the increase of serum level of 25(OH)D3, that is, the incidence of thyroid nodules in the high-level of 25(OH)D3 was lower than the low-level of 25(OH)D3. Nathan et al.’s study revealed that deficiency of vitamin D was found in both thyroid cancer and thyroid nodules, however, there was no significant difference in the degree of deficiency of vitamin D between thyroid cancer and thyroid nodules [16]. However, a number of scholars believe that serum 25(OH)D3 level cannot be used as a monitoring index for nodular thyroid disease [17]. Although there are few reports concerning the relationship between vitamin D and thyroid nodules, previous studies have found that deficiency of vitamin D is a risk factor for thyroid disease, which is consistent with our results as well. Consequently, we speculated that vitamin D may be reduced in the production of thyroid nodules by inhibiting cell proliferation, and a large number of controlled studies should be conducted in the future.

A study on children with simple goiter showed that serum IGF-1 levels in children with goiter were lower than those children without goiter, reflecting that low-levels of IGF-1 in serum may be associated with thyroid enlargement [18]. The Völzke et al.’s study [19] disclosed that the occurrence of thyroid nodules was associated with the high level of IGF-1 in serum, however, that was only observed in male patients. The results of this study showed that the serum IGF-1 level in the nodular group was lower than that in the control group. Logistic regression analysis showed that 25(OH)D3, IGF-1, FBG, TC, TT3, TT4, and high blood pressure were associated with thyroid nodules as well. Correlation analysis suggested that serum IGF-1 was positively correlated with serum 25(OH)D3, and negatively correlated with fasting blood glucose. Therefore, it is speculated that these factors may affect the relationship between serum IGF-1 and the incidence of thyroid nodules. Similarly, all the subjects were divided into four groups according to the four digits of serum IGF-1, then the incidence of thyroid nodules was reduced in Q1 group to Q4 group. Compared with Q1 group, the incidence of thyroid nodules in Q4 group decreased, and there was statistically significant difference (*P < 0.05*). After the aforementioned factors were corrected, the occurrence risk of thyroid nodules was no longer decreased with the increase of serum IGF-1 level. An investigation showed that IGF-1 may play an important role in the pathogenesis of certain thyroid cold nodules [20]. Hsiao et al. [21] compared the serum IGF-1 of the thyroid cold and hot nodule group with the control group, and found that the concentration of serum IGF-1 in the hot nodule group or the cold nodule group was not significantly different from the control group (*P* > 0.05). To date, the relationship between serum IGF-1 level and thyroid nodule is not still clear. In this study, the incidence of thyroid nodules was not decreased with the increase of serum IGF-1 levels after correction of those aforementioned factors. Therefore, we speculated that IGF-1 may be involved in the pathophysiological process of thyroid nodules along with 25(OH)D3 and FBG, however, further studies are required to confirm that finding.

There was no significant correlation between serum 25(OH)D3 or IGF-1 and the cross sectional area of thyroid nodules in this study, suggesting that serum 25(OH)D3 or IGF-1 may be independent of the size of thyroid nodules. LRA showed that serum 25(OH)D3 and IGF-1 were positively correlated with thyroid nodules. Correlation analysis showed that serum 25(OH)D3 was positively correlated with IGF-1. Therefore, we speculated that there may be some associations between serum 25(OH)D3 and IGF-1 in the formation of thyroid nodules. At present, there are relatively few reports concerning the relationship between serum 25(OH)D3, IGF-1, and thyroid nodules, however, similar reports have been found in some other diseases as well. A survey of type 2 diabetic patients showed that serum IGF-1 and other components were involved in the inflammatory process of diabetic nephropathy through vascular endothelial injury and inhibition of neovascularization. Serum 25(OH)D3 may be partially protected by inhibition of inflammation (e.g., inhibition of abnormal angiogenesis and vascular endothelial dysfunction [22]). Besides, a previous study conducted in Italy revealed that circulation of IGF-1 is a major vascular protection factor at low circulation levels of vitamin D, and the mechanism is that IGF-1 strengthens the antioxidant and anti-apoptotic effects in endothelial cell culture [23]. Therefore, it can be concluded that the role of IGF-1 in disease is different. The results of this study suggest that serum 25(OH)D3 is a protective effect of thyroid nodules, and IGF-1 may affect the occurrence of thyroid nodules by 25(OH)D3, meanwhile serum IGF-1 may be as one of the indirect protective factors for thyroid nodules,which needs further study. There were also some limitations in the course of this study: 1) the study did not consider the age range of the subjects, 2) the pathological typing of thyroid nodules was not performed in this study, and 3) the ultrasound results were not recorded in detail and the relevant statistical analysis was not performed.

## Conclusions

In summary. The incidence of thyroid nodules is relatively low in the high serum levels of 25(OH)D3, and serum 25(OH)D3 may be a direct protective factor for thyroid nodules. Serum IGF-1 may be as one of the indirect protective factors for thyroid nodules as well.

## Additional file

Additional file 1: Questionnaire. (PDF 64 kb)

## Abbreviations

25(OH)D3: Serum 25 hydroxyvitamin D3
ALT: Alanine aminotransfease
AST: Aspartate aminotransferase
BMI: Body mass index
FBG: Fasting blood glucose
FINS: Fasting insulin
HDL-C: High density lipoprotein cholesterol
HOMA-IR: Homeostasis model of assessment for insulin resistence index
IGF-1: Insulin-like growth factor-1
LDL-C: Low density lipoprotein cholesterol
LRA: Logistic regression analysis
r-GT: r-Glutamyl transferase
TC: Total cholesterol
TG: Triglyceride
TPOAb: Thyroid peroxidase antibody
TSH: Thyrotropin, thyroid stimulating hormone
TT3: Total triiodothyronine
TT4: Total thyroxine
WC: Waist circumference
