# Supplementary material for: Changes of serum 25(OH) D3 and IGF-1 levels in patients with thyroid nodules
Source: BMC Endocr Disord. 2019 May 10;19:48. doi: 10.1186/s12902-019-0376-1 (PMC6509827; doi:10.1186/s12902-019-0376-1)
Supplement: Supplementary file 1 — Questionnaire. (PDF 64 kb) [file 12902_2019_376_MOESM1_ESM.pdf]

Code: ____________

Questionnaire

First of all, thank you very much for participating in this survey. After the previous thyroid ultrasound examination, you are prompted (with ☐ / without ☐) thyroid nodules. In order to further study the mechanism of thyroid nodules, we will investigate your general information. The contents of the questions for the preliminary screening table must be filled out in detail, and 8-9 questions are measured and recorded by our professionals. Thank you again for your cooperation.

____________________District____________________City____________________Province

Contact number: ____________________ Email: ____________________

1. Name: ____________________
2. Gender: 1 = male ☐, 2 = female ☐
3. Ethnicity: 1 = Han ☐, 2 = other ☐
4. Date of birth (day-month-year) : ________________
5. Do you smoke (smoking criterion is more than one cigarette per day and continuous or cumulative for more than 6 months)? 0=Never☐(Jump to question 6), 1=Former☐, 2=Current☐

5.1 If you are still smoking or have ever smoked, how many cigarettes daily?______

5.2 If you are still smoking or have smoked, deduct the number of years of smoking cessation, how many years have been smoked (less than one year is calculated in one year)?______

6. Do you drink alcohol (often drinking refers to an average of at least once a week for more than 6 months)? 0=Never☐(Jump to question 7), 1=Former☐, 2=Sometimes☐, 3=Often☐

6.1 If you have already abstained from alcohol, how many years has this alcoholism been going on (less than one year is calculated in one year)?______

7. Past history: (Play "√" in the corresponding grid)

Past history		Have	No
History of thyroid disease	Hyperthyroidism		
	Hypothyroidism		
	Thyroid nodules		
Diabetes			
Hypertension			
Malignant tumor (specific tumor name)			
Acromegaly			
Liver and kidney disease (specific disease name)			
Other (recent history of the disease)			

NOTE: Diagnostic criteria for hypertension: systolic blood pressure ≥140 mmHg and/or diastolic blood pressure ≥90 mmHg.

8. Systolic blood pressure: ______mmHg, diastolic blood pressure:______mmHg
9. Height: ________cm, weight: ________kg, waist circumference: _______cm
10. Further research involves blood tests. You (agree☐/disagree☐) further participate. If you participate, the clinician will communicate with you later to sign an informed consent form; if you do not participate, we would like to thank you for participating in this survey.

Surveyor:_________________ Recorder:_________________

Date of completion (day-month-year): _______________
